# Effects of floor- and net-rearing systems on intestinal growth and microbial diversity in the ceca of ducks

**DOI:** 10.1186/s12866-022-02478-1

**Published:** 2022-03-16

**Authors:** Xuefei Chen, Liansi Huang, Lumin Cheng, Bo Hu, Hehe Liu, Jiwei Hu, Shenqiang Hu, Chunchun Han, Hua He, Bo Kang, Hengyong Xu, Jiwen Wang, Liang Li

**Affiliations:** Farm Animal Genetic Resources Exploration and Innovation Key Laboratory of Sichuan Province, Sichuan Agricultural University, Chengdu, Sichuan People’s Republic of China

**Keywords:** Duck, Rearing system, Intestinal growth, Ceca microorganisms

## Abstract

**Background:**

Rearing systems can affect livestock production directly, but whether they have effects on intestinal growth states and ceca microorganisms in ducks is largely unclear. The current study used Nonghua ducks to estimate the effects of rearing systems on the intestines by evaluating differences in intestinal growth indices and cecal microorganisms between ducks in the floor-rearing system (FRS) and net-rearing system (NRS).

**Results:**

The values of relative weight (RW), relative length (RL) and RW/RL of the duodenum, jejunum, ileum and ceca in the FRS were significantly higher than those in the NRS during weeks 4, 8 and 13 (*p* < 0.05). A total of 157 genera were identified from ducks under the two systems, and the dominant microorganisms in both treatments were Firmicutes, Bacteroidetes, Actinobacteria and Proteobacteria at the phylum level. The distribution of microorganisms in the ceca of the two treatments showed significant separation during the three time periods, and the value of the Simpson index in the FRS was significantly higher than that in the NRS at 13 weeks (*p* < 0.05). Five differential microorganisms and 25 differential metabolic pathways were found in the ceca at week 4, seven differential microorganisms and 25 differential metabolic pathways were found in the ceca at week 8, and four differential microorganisms and two differential metabolic pathways were found in the ceca at week 13.

**Conclusions:**

The rearing system influences duck intestinal development and microorganisms. The FRS group had higher intestinal RL, RW and RW/RL and obviously separated ceca microorganisms compared to those of the NRS group. The differential metabolic pathways of cecal microorganisms decreased with increasing age, and the abundance of translation pathways was higher in the NRS group at week 13, while cofactor and vitamin metabolism were more abundant in the FRS group.

## Background

Digestion and nutrient absorption are the basic functions of the intestines and mainly occur in the small intestine, which is also the longest part of the digestive tract. The mucosa is a crucial component of the small intestinal wall that includes many finger-like villi extending from the mucosal layer into the lumen, increasing the surface area of the small intestine by 600 times compared to that of the whole intestinal cavity, and nutrients in intestinal contents can be easily absorbed because the villi contain tightly packed blood capillaries with thin vascular walls [15, 24, 33]. The nutrient digestive and absorptive capacity of the intestinal tract depends on the comprehensive action of the pancreas, intestinal enzyme activity, intestinal surface area and intestinal nutrient transport carriers [20, 32], and the surface area of intestinal villi is the key factor limiting the growth of poultry [26, 40]. Thus, increasing the length and weight of the intestinal tract helps expand the food-digestion area and promote the digestion and absorption of nutrients.

Intestinal microorganisms are known as the “second genome” of the host, and approximately 35% of microbial enzymes in the intestine can be utilized by the host. Intestinal microorganisms play important roles in body growth and health by impacting intestinal villus and crypt morphology, nutrient metabolism regulation, mucosal immune activation, energy-rich short-chain fatty acid production, host behavior regulation, intestinal epithelial cell repair and pathogenic microorganism resistance [9, 11, 14, 23, 37]. Food rapidly passes through the front of the intestinal tract but stays in the end of the tract for several hours [19]. The ceca, as the main site of intestinal microbial colonization and the main area of microbial anaerobic fermentation with the highest content of short-chain fatty acids, has a higher fermentation ability than the small intestine [7], is considered the most important to poultry health and is a major pathogen reservoir [30, 31, 38]. The abundance and diversity of ceca microorganisms are influenced by many factors [8, 13, 25], and rearing systems are an important factor. The microbial diversity in the ceca of wild red-crowned cranes is lower than those of captive and artificially raised cranes, and the microorganismal composition is also significantly variable [43], which is consistent with results in Kakapo parrots, Antarctic seals and wild-captured rodents [18, 36]. However, in contrast to the above findings, the microbial abundance in the ceca of Dagu chickens raised outdoors is higher than that of cage-raised chickens [44].

After China’s inclusion in the WTO, the country’s export share of duck primary products and byproducts, such as duck meat and duck eggs, greatly increased. According to FAO statistics, the number of ducks raised and stocked in China have ranked first in the world in recent years. The floor-rearing system (FRS) and net-rearing system (NRS) are the two main systems of intensive duck production. The FRS is the most primitive method of duck farming in China due to its low cost and high muscle growth and product quality, while the NRS allows excreta to be removed through metal nets, thus keeping the rearing environment clean [1]. However, the FRS requires a particular rearing area and frequent replacement of cushions, which are challenges, and diseases easily occur in FRSs due to direct contact with feces. Currently, most farmers build grid structures approximately 60 cm above the ground and install metal nets to remove excreta, but the cost is relatively high, and the cleaning and disinfection of the nets are inconvenient. In this study, we aimed to perform a comprehensive assessment of intestinal growth and microorganisms in the ceca of ducks in an FRS and an NRS. The results of this study will offer useful information for selecting an appropriate and healthy rearing systems for ducks and provide a theoretical and practical reference for the further study of duck rearing systems.

## Results

### Effects of the rearing system on the growth of the small intestine

The relative weight (RW)/relative length (RL) ratios of the duodenum, jejunum, and ileum and the jejunal RW in the FRS group were significantly higher than those in the NRS at 4 weeks (*p* < 0.05), and the cecal RW and RW/RL were significantly higher in the FRS group (*p* < 0.01). The RW/RL of the ceca in the FRS group was significantly higher than that in the NRS group at 8 weeks (*p* < 0.05), and all other intestinal growth-related indices, including the RL, RW and RW/RL, in the FRS group were also significantly higher (*p* < 0.01). The RW/RL and RW values of the small intestine and the RL of the ileum in the FRS group remained significantly higher than those in the NRS group at 13 weeks (*p* < 0.01), and the cecal RW/RL in the NRS group was also significantly lower than that in the FRS group (*p* < 0.05). In addition, the body weights of ducks in the two systems were also statistically analyzed, and it was found that compared to those in the NRS group, the body weights in the FRS group were significantly higher at 4 weeks (*p* < 0.05) but significantly lower at 8 weeks (*p* < 0.05). However, there was no significant difference in body weight between the two systems at week 13 (*p* > 0.05) (Table 1).

**Table 1:** Effects on the body weight and intestinal growth of ducks

Week	System	Body	Segment	System	Relative	Relative	Relative Weight/
		Weight			Length	Weight	Relative Length
		(kg)			(cm/kg)	(g/kg)	(cm/g)
4	FRS	1.07 ± 0.12	Duodenum	FRS	26.05 ± 1.86	4.34 ± 0.34	0.17 ± 0.02
	NRS	1.02 ± 0.10		NRS	26.01 ± 1.35	4.16 ± 0.66	0.15 ± 0.02
	*P value*	0.03		*P value*	0.90	0.12	0.02
			Jejunum	FRS	63.59 ± 4.08	11.48 ± 1.09	0.18 ± 0.02
				NRS	63.88 ± 3.09	10.86 ± 1.43	0.17 ± 0.02
				*P value*	0.71	0.03	0.02
			Ileum	FRS	60.78 ± 4.07	10.62 ± 0.78	0.18 ± 0.01
				NRS	61.38 ± 3.31	10.33 ± 0.99	0.17 ± 0.02
				*P value*	0.46	0.13	0.01
			Ceca	FRS	13.76 ± 1.43	1.80 ± 0.58	0.13 ± 0.05
				NRS	13.77 ± 1.06	1.20 ± 0.32	0.09 ± 0.02
				*P value*	0.98	0.00	0.00
8	FRS	2.11 ± 0.21	Duodenum	FRS	12.98 ± 0.88	2.66 ± 0.21	0.21 ± 0.02
	NRS	2.35 ± 0.24		NRS	11.94 ± 0.65	2.33 ± 0.18	0.20 ± 0.01
	*P value*	0.19		*P value*	0.00	0.00	0.00
			Jejunum	FRS	32.46 ± 3.32	6.31 ± 0.50	0.20 ± 0.01
				NRS	28.95 ± 1.62	4.97 ± 0.36	0.18 ± 0.01
				*P value*	0.00	0.00	0.00
			Ileum	FRS	31.68 ± 2.11	5.91 ± 0.34	0.19 ± 0.01
				NRS	27.77 ± 1.74	4.81 ± 0.37	0.18 ± 0.01
				*P value*	0.00	0.00	0.00
			Ceca	FRS	14.83 ± 1.09	1.43 ± 0.16	0.10 ± 0.01
				NRS	13.36 ± 0.86	1.23 ± 0.13	0.10 ± 0.01
				*P value*	0.00	0.00	0.03
13	FRS	2.39 ± 0.26	Duodenum	FRS	13.77 ± 1.06	2.29 ± 0.23	0.21 ± 0.01
	NRS	2.37 ± 0.26		NRS	10.91 ± 0.91	1.93 ± 0.23	0.18 ± 0.01
	*P value*	0.73		*P value*	0.28	0.00	0.00
			Jejunum	FRS	11.16 ± 0.82	5.50 ± 0.58	0.20 ± 0.01
				NRS	27.63 ± 2.55	4.19 ± 0.66	0.17 ± 0.01
				*P value*	0.11	0.00	0.00
			Ileum	FRS	27.59 ± 1.99	5.20 ± 0.56	0.19 ± 0.01
				NRS	25.82 ± 2.15	4.05 ± 0.68	0.17 ± 0.01
				*P value*	0.00	0.00	0.00
			Ceca	FRS	12.28 ± 1.06	1.27 ± 0.18	0.11 ± 0.01
				NRS	11.84 ± 1.00	1.25 ± 0.16	0.11 ± 0.01
				*P value*	0.10	0.59	0.03

*Note*: FRS represents the floor-rearing system, and NRS represents the net-rearing system. *n* = 30

### Effects of the rearing system on ceca microorganisms

**Fig. 1:**
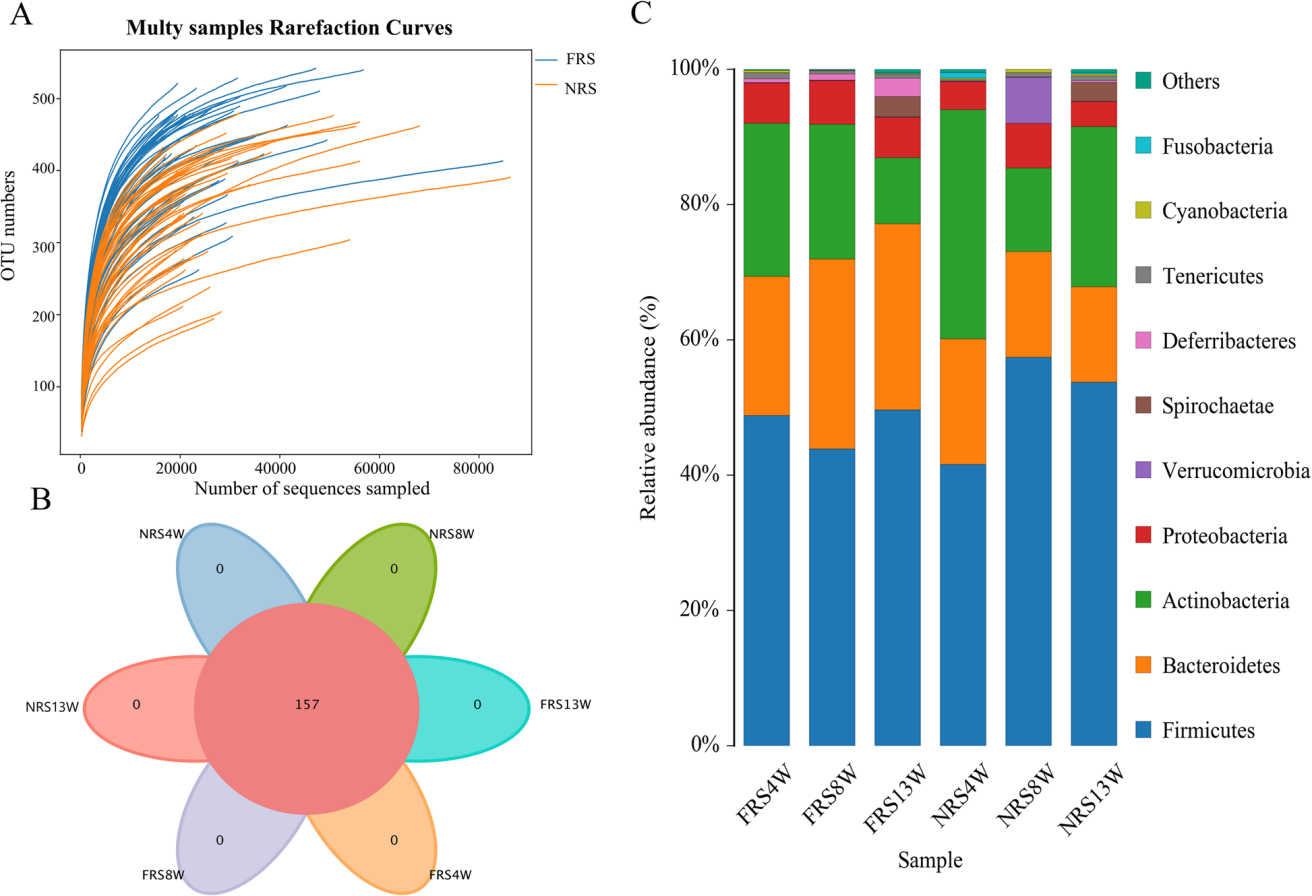
Bacterial community composition of kinds (genus level) and abundance (phyla level). **A** Multy sample rarefaction curves of microorganisms in cecal contents of ducks. **B** Venn map of cecal microorganisms at genus level at week 4, 8 and 13. **C** Distribution of cecal microorganisms at phylum level. All the microorganisms are expressed as percentages, and only the top 10 microbial phyla are shown. In **A**, **B** and **C**, FRS represents floor-reared systems, and NRS represents net-reared systems. 4 W, 8 W and 13 W represent 4 weeks of age, 8 weeks of age and 13 weeks of age

A total of 4,612,553 clean tags were generated from 104 samples of duck cecal contents after splicing and filtering for quality, and each sample produced at least 25,925 clean tags. The rarefaction curve of the number of OTUs based on sequencing tended to reach a saturation plateau, suggesting that 104 samples were adequate for estimating the phenotype richness and microbial community diversity of ceca microorganisms at a 97% similarity threshold, and broadly, the microbial abundance in the FRS group was higher than that in the NRS group (Fig. 1 A). To investigate the microbial community in the ceca of the FRS and NRS groups, pairwise comparisons of microbial similarity between the two systems were performed, and analyses of the common and unique OTUs were conducted. A total of 157 genera were identified from ducks in the two systems. However, no specific microorganisms were found in any intestinal segment at 4, 8 or 13 weeks (Fig. 1 B). The bacterial phyla of the top ten most abundant microorganisms in the ceca were determined, and the dominant microorganisms in both treatments were Firmicutes (43.87% ~ 49.61% vs. 41.58% ~ 57.40%), Bacteroidetes (20.54% ~ 28.06% vs. 14.14% ~ 18.51%), Actinobacteria (9.79% ~ 22.67% vs. 12.34% ~ 33.89%) and Proteobacteria (5.93% ~ 6.41% vs. 3.66% ~ 6.58%). The abundance of Bacteroidetes at 13 weeks was higher than that of Actinobacteria in the FRS group (27.56% vs. 9.79%), and the exact opposite result was found in the NRS group (14.14% vs. 23.69%) (Fig. 1 C).

**Fig. 2:**
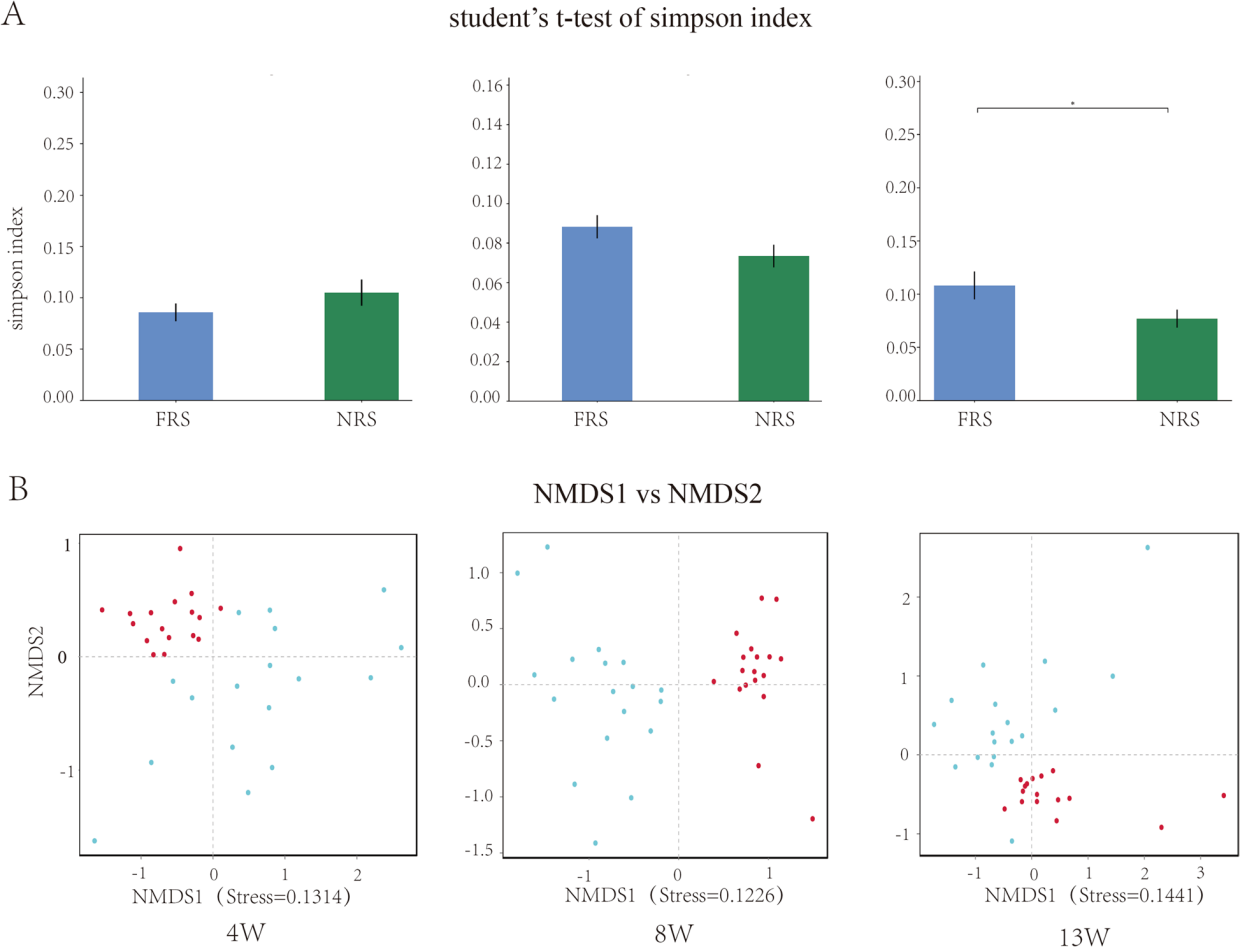
Analysis of microbial diversity. **A** Simpsons index at genus level of microorganisms in cecal contents of ducks. **B** NMDS analysis of cecal microorganisms at genus level. In **A** and **B**, FRS represents floor-reared systems, and NRS represents net-reared systems. 4 W, 8 W and 13 W represent 4 weeks of age, 8 weeks of age and 13 weeks of age

The Simpson index showed no significant difference between the FRS and NRS groups at 4 and 8 weeks based on analyzing the microbial diversity of the cecal contents (*p* > 0.05), while the value in the FRS group was significantly higher than that in the NRS group at 13 weeks (*p* < 0.05) (Fig. 2 A). In addition, the distributions of cecal microorganisms in the two rearing systems were obviously separated during the three time periods of the experiment (stress 1 = 0.1314, stress 1 = 0.1226, and stress 1 = 0.1441) (Fig. 2 B). LEfSe analysis was carried out to determine the specific microorganisms responsible for microorganism diversity at the species level. At 4 weeks, the abundances of *Ruminococcaceae* uncultured bacterium, *Ruminococcaceae UCG-014* and *Desulfovibrio* were higher in the FRS group than in the NRS group, while the *Brachybacterium* and *Lactobacillus* genera had higher abundances in the NRS group. At 8 weeks, the genera *Brevibacterium*, *Brachybacterium* and *Bacteroides* were enriched in the FRS group, while *Subdoligranulum*, *Akkermansia*, *Blautia* and *Collinsella* were enriched in the NRS group. At 13 weeks, the abundances of the genera *Bacteroides* and *Ruminococcaceae* uncultured bacterium were higher in the FRS group, while *Subdoligranulum* and *Brachyspira* were more abundant in the NRS group (Table 2).

**Table 2:** LEfSe analysis of the ceca microorganisms

Week	Microorganism	Abundance	System	LDA	*p value*
4	Lactobacillus	4.63	NRS	4.08	0.03
	Desulfovibrio	4.71	FRS	4.21	0.00
	Brachybacterium	4.91	NRS	4.29	0.01
	uncultured_bacterium_f_Ruminococcaceae	4.63	FRS	4.03	0.01
	Ruminococcaceae_UCG_014	4.41	FRS	4.06	0.00
8	Bacteroides	5.32	FRS	4.72	0.00
	Collinsella	4.66	NRS	4.19	0.00
	Blautia	4.59	NRS	4.02	0.00
	Akkermansia	4.82	NRS	4.57	0.00
	Subdoligranulum	4.93	NRS	4.14	0.02
	Brevibacterium	4.77	FRS	4.45	0.00
	Brachybacterium	4.88	FRS	4.57	0.00
13	Brachyspira	4.58	NRS	4.18	0.02
	Bacteroides	5.3	FRS	4.75	0.00
	Subdoligranulum	4.99	NRS	4.41	0.01
	uncultured_bacterium_f_Ruminococcaceae	4.92	FRS	4.39	0.00

*Note*: FRS represents the floor-rearing system, and NRS represents the net-rearing system

**Fig. 3:**
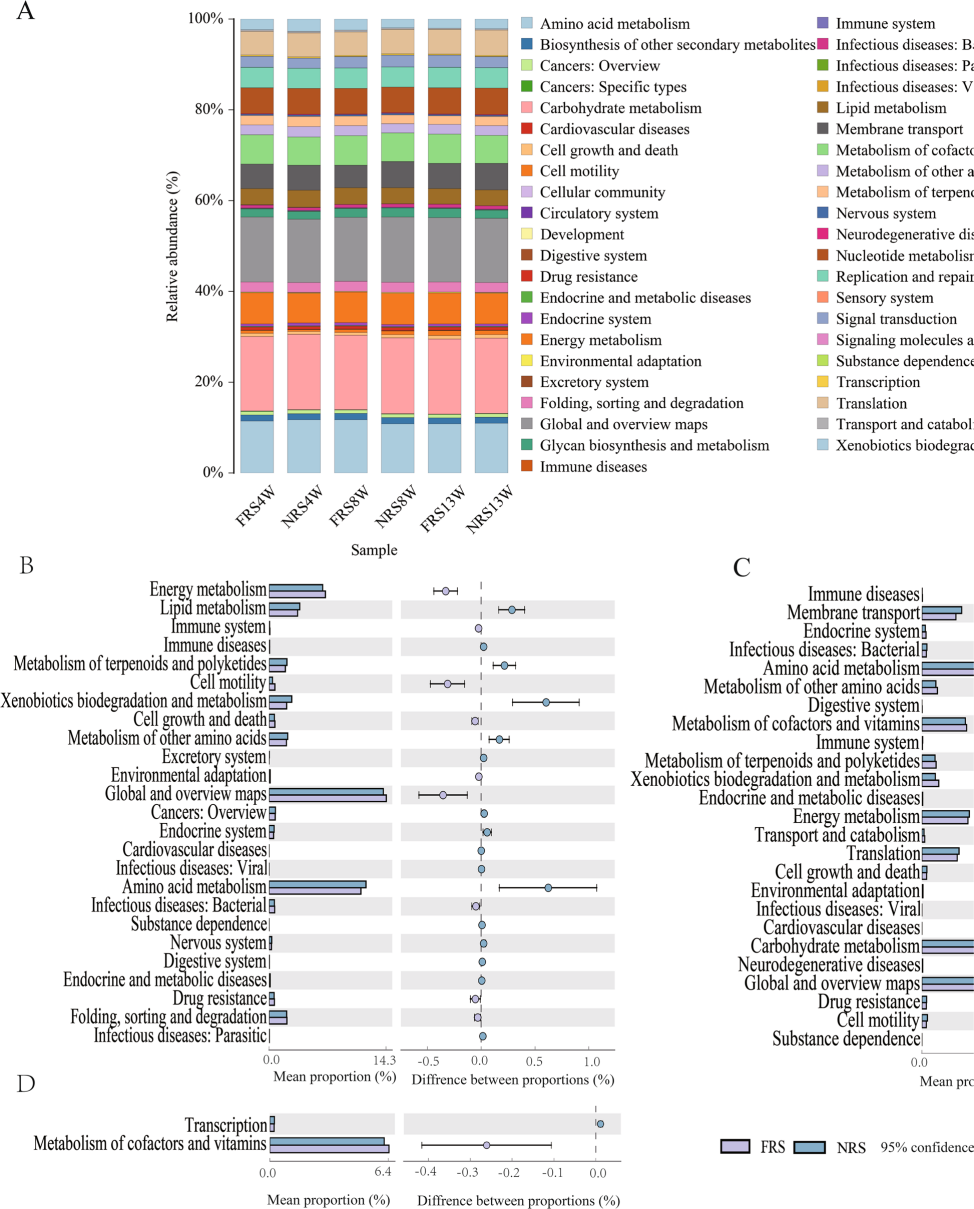
KEGG pathway comparison. **A** Distribution of functional pathways of microorganisms in cecal contents of ducks. All the microorganisms are expressed as percentages. **B** Differential function pathways at 4 weeks. **C** Differential function pathways at 8 weeks. **D** Differential function pathways at 13 weeks. In **A, B**, **C** and **D**, FRS represents floor-reared systems, and NRS represents net-reared systems. 4 W, 8 W and 13 W represent 4 weeks of age, 8 weeks of age and 13 weeks of age

Further analysis of the microorganisms in the ceca was conducted to study the functional pathway differences at the class-two level among the FRS and NRS groups, and the results showed that carbohydrate metabolism (16.36% ~ 16.59% vs. 16.52% ~ 16.72%), global and overview maps (14.00% ~ 14.29% vs. 14.14% ~ 14.37%) and amino acid metabolism (11.46% ~ 11.71% vs. 10.80% ~ 10.93%) were the top-three most abundant in both systems (Fig. 3 A). There were 25 significantly different pathways between the two systems at 4 weeks, and nine pathways were more abundant in the FRS group, including drug resistance, environmental adaptability, energy metabolism and cell motility pathways (Fig. 3 B). Similar to the results at 4 weeks, 25 different pathways, including 11 functional pathways, such as immune diseases, cofactor and vitamin metabolism, endocrine system and amino acid metabolism, were more abundant in the FRS group at 8 weeks (Fig. 3 C). However, only cofactor and vitamin metabolism pathways were more abundant in the FRS group at 13 weeks, while the abundance of translation pathways was higher in the NRS group (Fig. 3 D).

## Discussion

The host and environment both influence the microorganisms of the ceca [4, 12]. The FRS and NRS are two main methods used in intensive farming of ducks. The growth of the small intestine in outdoor environments, such as grazing areas and artificial grasslands, was significantly higher than that in caged environments [3, 44], and a similar situation was found in this experiment. Both the intestinal relative length, which can reflect the intestinal capacity, and ratio of relative weight to relative length, which reflects intestinal motility, were higher in the FRS group, suggesting that ducks reared in FRSs have stronger intestinal peristalsis ability and larger food digestion areas, which may result from increased activity in swimming pools. Many reports have shown that the length and weight of poultry intestines are affected by the level of fiber intake; for example, dietary sunflower hulls could increase the length of the small intestine in broilers [17]. Considering the specific situation in this experiment, this result may be because in addition to artificial diets, ducks in the FRS also consume mattresses on the ground and algae in ponds, which increases their fiber levels. Therefore, it can be concluded that FRSs are more conducive to duck intestinal growth than NRSs.

The comprehensive characterization of duck intestinal microbial communities is a critical precondition for understanding and predicting how rearing systems alter these communities. To further explore the differences between the FRS and NRS, the sequences of the ceca contents were detected. Although there were no cecal microorganisms specific to ducks in either system at the genus level, the diversity showed a significant difference at 13 weeks. In general, the diversity of the intestinal microbial composition of poultry gradually increases with increasing age after birth. All kinds of microorganisms increase and decrease in abundance over time after birth and then tend to reach a relatively stable state in youth [25]. Therefore, the difference in diversity of duck intestinal microorganisms in this study is convincing. Intestinal microorganisms are affected by many factors, including age, sex and the environment [10, 16, 27]. The different rearing systems in this study provided different growth environments for ducks, with increased diversity observed in ground-based ducks, which was consistent with the result found in Dagu chickens [44]. Considering that long-term stress can reduce the diversity of intestinal microbiota [2], this result may be because the ducks in the NRS were raised on a net and unable to contact the natural environment. Firmicutes, Bacteroidetes, Actinobacteria and Proteobacteria were dominant in the ceca in both systems, and this result coincides with those in wild turkeys, captive broilers, caged Beijing ducks and floor-raised Landes geese [21, 22, 34, 42].

At 4 weeks, the abundance of environmental adaptability pathways of microorganisms in the ceca of ducks in the FRS group was higher, and that of metabolic-related pathways, including xenobiotic biodegradation and metabolism, amino acid metabolism and lipid metabolism, was lower, suggesting that the change in the living environment after the brooding period caused stress. The abundances of most pathways related to diseases, including cardiovascular disease pathways, substance dependence and viral infectious diseases, in the NRS group were higher than those in the FRS group at 4, 8 or 13 weeks, and these diseases cause serious harm to the body, which is consistent with previous studies [39, 41]. Of the eight metabolic pathways examined at 8 weeks, the abundances of five were greater in the FRS group than in the NRS group, suggesting that ducks in the FRS had adapted to the environment and ingested more substances, improving the meat quality. Studies have shown that the meat of outdoor chickens is darker and has a better water-holding capacity [5, 35]. In addition, of the 25 functional pathways identified at 8 weeks, 16 pathways were also found at 4 weeks, and their abundances changed between 4 weeks and 8 weeks. At 13 weeks, only the cofactor and vitamin metabolism pathways in the FRS group were more abundant due to the higher abundance of *Bacteroides* and *Ruminococcaceae* uncultured bacterium, while the abundance of translation pathways in the NRS group was higher due to the presence of *Subdoligranulum* and *Brachyspira*, implying that the differences in functional pathways of microorganisms in the ceca of ducks between the FRS and NRS groups gradually decreased over time. As discussed previously, the colonization of intestinal microflora is a process that changes with age [29]; thus, differential metabolic pathways and microorganisms also undergo a gradual stabilization process. When ducks enter the youth period and the intestinal environment becomes relatively stable, the influence of the FRS and NRS systems on duck intestinal development and microorganisms can be revealed.

## Conclusion

There were differences in intestinal development and microorganisms between ducks in the floor-rearing system (FRS) and net-rearing system (NRS). The intestinal relative length, relative weight and relative weight/relative length in the FRS group were higher than those in the NRS group. The ceca microorganisms of ducks in the two rearing systems were obviously separated, and the diversity of cecal microorganisms was higher in the FRS group at 13 weeks. The differential metabolic pathways of cecal microorganisms decreased with increasing age, and the abundance of translation pathways was higher in the NRS group at week 13, while cofactor and vitamin metabolism pathways were more abundant in the FRS group.

## Methods

### Laboratory animals and sample collection

The Nonghua ducks used in this experiment were provided by the poultry-raising experimental farm of Sichuan Agricultural University. Healthy ducks of the same weight were randomly divided equally into the floor-rearing system (FRS) and net-rearing system (NRS) groups after brooding, with a stocking density of 4–5/m^2^. Each system uses three grids for repetitions, and each grid hold 30 ducks. The pen house adopted a semiopen design that included two parts—the egg-laying rest area in the house and the outdoor sports field, the areal ratio of which is approximately 1:3. The outdoor sports field is composed of the ground sports field and the water sports field. The house is maintained in thick-litter mode, and the outside is flushed every day, while the pool water is replaced every three to five days. The NRS has four square meters per grid, and each grid raises approximately 25 ducks. The intake of feed and water by each duck was ensured ad libitum during the experiment, and all ducks were subjected to the same routine immunization procedures to meet the National Research Council requirements. Table 3 shows the nutritional standards for the different stages of ducks [6]. Each treatment randomly selected 30 healthy male ducks at 4, 8 and 13 weeks, respectively (180 in total), weighed and euthanized by cervical dislocation after fasting for 12 h. After dissection, the duodenum, jejunum, ileum and ceca were removed, and the cecal contents were collected and quick-frozen in liquid nitrogen. All animal handling mentioned were reviewed and approved by the Animal Ethics Committee of Sichuan Agricultural University (Ya’an, China).

**Table 3:** Dietary nutritional standards for different stages of ducks

Nutrition	Week	
	0 ~ 3	3 ~ 13
Moisture (%)	≤14.0	≤14.0
Crude protein (%)	≥19.0	≥15.0
Crude fiber (%)	≤6.0	≤7.0
Coarse ash (%)	≤8.0	≤10.0
Calcium (%)	0.8 ~ 1.5	0.8 ~ 1.5
Total phosphorus (%)	≥0.60	≥0.60
Sodium chloride (%)	0.3 ~ 0.8	0.3 ~ 0.8
Methionine (%)	≥0.35	≥0.30

### Measurement of intestinal growth

The tissues of the duodenum, jejunum, ileum and ceca, including the pancreas and fat attached to the intestine, were removed. Then, one end of the intestine was fixed onto a glass plate wetted with distilled water and gently straightened to measure the length of the duodenum, jejunum, ileum and ceca when the intestine was no longer retracted. The contents of the intestine were removed, and the aforementioned four intestinal segments were weighed with an electronic balance. The relative length (RL), relative weight (RW) and RW/RL were calculated.

$$\mathrm{RL}=\mathrm{intestinal}\ \mathrm{length}\ \left(\mathrm{cm}\right)/\mathrm{live}\ \mathrm{weight}\ \left(\mathrm{kg}\right)$$

$$\mathrm{RW}=\mathrm{intestinal}\ \mathrm{weight}\ \left(\mathrm{g}\right)/\mathrm{live}\ \mathrm{weight}\ \left(\mathrm{kg}\right)$$

### DNA extraction

A Power Soil DNA Isolation Kit (MO BIO Laboratories) was used to extract the total bacterial DNA of the ceca content and stored at −80 °C for further processing. The ratios of 260 nm/280 nm and 260 nm/230 nm absorbence were used as indicators to evaluate DNA quality and quantity.

### Determination of ceca microorganisms

5′-ACTCCTACGGGAGGCAGCA-3′ (forward primer) and 5′-GGACTACHVGGGTWTCTAAT-3′ (reverse primer) were used to combine the adapter sequences and barcode sequences to obtain the bacterial 16S rRNA gene V3-V4 region. Ten microliters of buffer, 10 μL of high-GC enhancer, 10 μM of each primer, 0.2 μL of Q5 high-fidelity DNA polymerase, 1 μL of dNTPs, and 60 ng of genomic DNA were mixed for PCR amplification. The thermal cycling conditions were as previously reported [28]. The PCR products were then subjected to a second round of purification using Agencourt Ampure XP beads (Beckman, USA) with 8 μL ddH_2_O, 20 μL 2× Phμsion HF MM, 10 μM of each primer, and 10 μL products from the first round of PCR. The thermal cycling conditions were as follows: 98 °C for 30 s, 98 °C for 10 s for 10 cycles, 65 °C for 30 s, 72 °C for 30 s, and extension at 72 °C for 5 min. The PCR products were quantified using the PicoGreen dsDNA Assay kit (Invitrogen, USA) and equally combined, followed by gel purification using a QIAquick Gel Extraction Kit (Qiagen, USA) and requantification with PicoGreen. The Illumina HiSeq 2500 platform (2 × 250 paired ends) was used for sequencing. Sequences overlapping by more than 10 bp were assembled by FLASH (version 1.2.11), filtered via Trimmomatic (version 0.33), and became high-quality tag sequences after removing chimeras using UCHIME (version 8.1). Operational taxonomic units (OTUs) were generated using the Uparse clustering method (97% cutoff) (USEARCH, version 10.0), and all samples were rarefied to the same sequencing depth by resampling the OTUs prior to downstream analysis.

### Data statistic analysis

Mothur software (version v.1.30) and QIIME software (version 1.8) were used to evaluate the alpha diversity and beta diversity of the samples. The beta diversity distance matrix was calculated by QIIME (version 2), and differences were reflected on a two-dimensional coordinate map for NMDS analysis. With biomarkers obtained based on LDA > 4, Kruskal–Wallis and Wilcoxon rank sum test were used to detect differences in LEfSe analysis (version 1.5.3). The figures were drawn using the R language tool (version 3.6.0). SPSS 21.0 software (IBM, USA) was used to analyze the data, and a t test was used to analyze the significance of the sample data. The data are expressed as the mean ± S.D. Statistically, *p* < 0.05 represents a significant difference, and *p* < 0.01 indicates an extremely significant difference.

### Availability of data and materials

The datasets analyzed during the current study are available in the Genome Sequence Archive under accession CRA006072 (https://bigd.big.ac.cn/gsa/browse/CRA006072).

## Data Availability

The datasets used and analyzed during the current study are available from the corresponding author upon reasonable request.

## Abbreviations

FRS: Floor-rearing system
NRS: Fet-rearing system
RL: Relative length
RW: Relative weight
OTUs: Operational taxonomic units
LEFse: Linear discriminant analysis effect size
